# Machine Learning for Coronary Heart Disease Prediction: Comparative Analysis of Framingham and Cleveland Subset of the UCI Dataset with SHAP-Based Interpretability

**DOI:** 10.3390/epidemiologia7030075

**Published:** 2026-06-01

**Authors:** Shreyas Raman, Devansh Thakkar, Jacques Calixte, Rahul Kumar, Kyle Sporn, Kiran Marla, Divyam Goel, Rhea Gopali, Nitin Chetla, Saif Pasha, Nikitha Ravisankar, Ryung Lee, Ciprian Ionita

**Affiliations:** 1 Khoury College of Computer Sciences, Northeastern University, Boston, MA 02115, USA; shreyasraman53@gmail.com (S.R.); devanshthakkar1980@gmail.com (D.T.); 2 Vagelos College of Physicians and Surgeons, Columbia University, New York, NY 10032, USA; jc5968@cumc.columbia.edu; 3 Department of Medicine, University of Massachusetts Chan School of Medicine, Worcester, MA 01655, USA; 4 Department of Medicine, Norton College of Medicine, SUNY Upstate Medical University, Syracuse, NY 13210, USA; 5 Carver College of Medicine, University of Iowa, Iowa City, IA 52242, USA; kmarla@uiowa.edu; 6 Department of Medicine, Duke University School of Medicine, Durham, NC 27710, USA; divyam.goel@duke.edu; 7 School of Osteopathic Medicine, Campbell University School of Osteopathic Medicine, Lillington, NC 27546, USA; rsgopali0520@email.campbell.edu; 8 Department of Radiology, University of Virginia, Charlottesville, VA 22903, USA; 9 Department of Medicine, Virginia Tech Carilion School of Medicine, Roanoke, VA 24016, USA; 10 Department of Medicine, Jacobs School of Medicine and Biomedical Sciences, University at Buffalo, Buffalo, NY 14203, USA; 11 Department of Medicine, UB Clinical and Translational Research Center, University at Buffalo, Buffalo, NY 14203, USA

**Keywords:** machine learning, coronary heart disease, cardiovascular risk prediction, Framingham Heart Study, Cleveland subset, SHAP, interpretability

## Abstract

Introduction: Cardiovascular disease (CVD) remains the leading cause of mortality worldwide, with coronary artery disease (CAD), also known as ischemic heart disease (IHD), responsible for approximately 13% of global deaths in 2021. Studies applying machine learning (ML) and deep learning (DL) to heart disease classification have demonstrated promising results in risk prediction and feature extraction. Background/Objectives: In this study, we develop an AI/ML framework to predict and classify ischemic heart disease risk using publicly available datasets, the Framingham Heart Study and the Cleveland subset of the UCI Heart Disease dataset, along with explanations for how predictions were made by a process called SHAP (SHapley Additive exPlanations). Methods: We implemented a leakage-controlled machine learning pipeline that included data cleaning, stratified 80/20 train-test splitting, training-fold-only feature scaling and class balancing, 5-fold hyperparameter tuning, SHAP interpretability, and Brier score-based calibration assessment. Logistic regression, random forest, K-nearest neighbors, XGBoost, and a feedforward neural network were evaluated on the Framingham dataset and the Cleveland subset of the UCI Heart Disease dataset. Performance was assessed using accuracy, precision, recall, F1-score, Matthews correlation coefficient, AUROC, and Brier score. Results: After leakage-controlled evaluation, Framingham performance was more modest than in the preliminary analysis. Logistic regression achieved the highest AUROC on the Framingham dataset (0.7234), while random forest achieved the lowest Brier score (0.1750), and the feedforward neural network achieved the highest accuracy (0.7719). On the Cleveland subset, logistic regression achieved the strongest threshold-based performance (accuracy 0.8667, precision 0.8571, recall 0.8571, F1-score 0.8571, MCC 0.7321), whereas K-nearest neighbors achieved the highest AUROC (0.9531) and lowest Brier score (0.0942). SHAP highlighted systolic blood pressure, smoking status, and hypertension as influential predictors (Framingham) and number of major vessels, chest pain type, thallium stress-test result (thal; normal, fixed defect, or reversible defect), and age (Cleveland) as top predictors. Conclusions: Optimal model performance is dataset-dependent, and SHAP enhances clinical interpretability. Broader access to high-quality, de-identified medical data could accelerate reproducible ML research in cardiology.

## 1. Introduction

Cardiovascular disease (CVD) remains the leading cause of mortality worldwide, with coronary artery disease (CAD), also known as ischemic heart disease (IHD), responsible for approximately 13% of global deaths in 2021 [1]. IHD occurs when blood flow to the heart is insufficient, often due to atherosclerotic stenosis, arterial blockages, or embolic events. Progression of CAD can ultimately lead to congestive heart failure (CHF), when the heart can no longer adequately pump blood. Other forms of non-ischemic heart disease, such as arrhythmias, congenital heart defects, valvular disease, cardiomyopathies, rheumatic heart disease, and endocarditis, present overlapping symptoms and often require advanced imaging or clinical context for diagnosis [2]. These conditions are underrepresented in publicly available datasets, whereas ischemic heart disease has been extensively studied, notably through the landmark Framingham Heart Study, which first established multivariable risk prediction models for CAD and laid the foundation for the widely used Framingham risk score [3].

Despite decades of epidemiologic research, public cardiac datasets remain limited in both scope and quality. Many available datasets provide only partial clinical information, lack longitudinal follow-up, or are imbalanced toward non-disease cases, restricting their utility for robust model development [4]. Studies applying machine learning (ML) and deep learning (DL) to heart disease classification have demonstrated promising results in risk prediction and feature extraction [5]. For example, a recent meta-analysis of 17 studies involving 285,213 patients reported strong performance of DL-based models (AUC ≈ 0.84), often comparable to or exceeding established methods such as random forests, support vector machines, and gradient boosting [6]. However, reproducibility and clinical interpretability remain key challenges, as many models rely on proprietary datasets or opaque feature representations (Table 1).

In this study, we develop an AI/ML framework to predict and classify ischemic heart disease risk using publicly available datasets, including the Framingham Heart Study and the Cleveland subset of the University of California, Irvine (UCI) Heart Disease dataset. Our approach emphasizes comprehensive preprocessing, including class imbalance handling and feature scaling, and incorporates model interpretability through SHAP analysis. By leveraging classical and modern machine learning techniques, we aim to identify the most predictive features for CAD and related conditions, enabling interpretable, data-driven risk assessment without dependence on specialized clinical imaging or proprietary datasets.

The novelty of this study lies not in proposing a new algorithm but in providing a structured cross-dataset comparison of commonly used machine learning models under a unified preprocessing, tuning, and interpretability framework. By evaluating model behavior across the Framingham Heart Study and the Cleveland subset of the UCI Heart Disease dataset, and applying SHAP consistently across datasets, this work highlights how predictive performance and feature-importance patterns can vary substantially by dataset characteristics. These findings emphasize the importance of cautious model selection and interpretation when translating machine learning methods across heterogeneous clinical datasets (Table 1).

## 2. Materials and Methods

This study was conducted using retrospective, publicly available data sets and did not involve direct interaction with human participants or the collection of identifiable personal data. As such, formal institutional review board (IRB) approval and informed consent were not required in accordance with local regulations and institutional policies. The study methodology complies with the principles outlined in the Declaration of Helsinki. All datasets were fully anonymized and publicly available prior to analysis, ensuring the protection of participant confidentiality and privacy. Details regarding dataset selection are posted in Supplementary Materials File (S1 and S2).

### 2.1. Data Preprocessing

**Figure 1:**
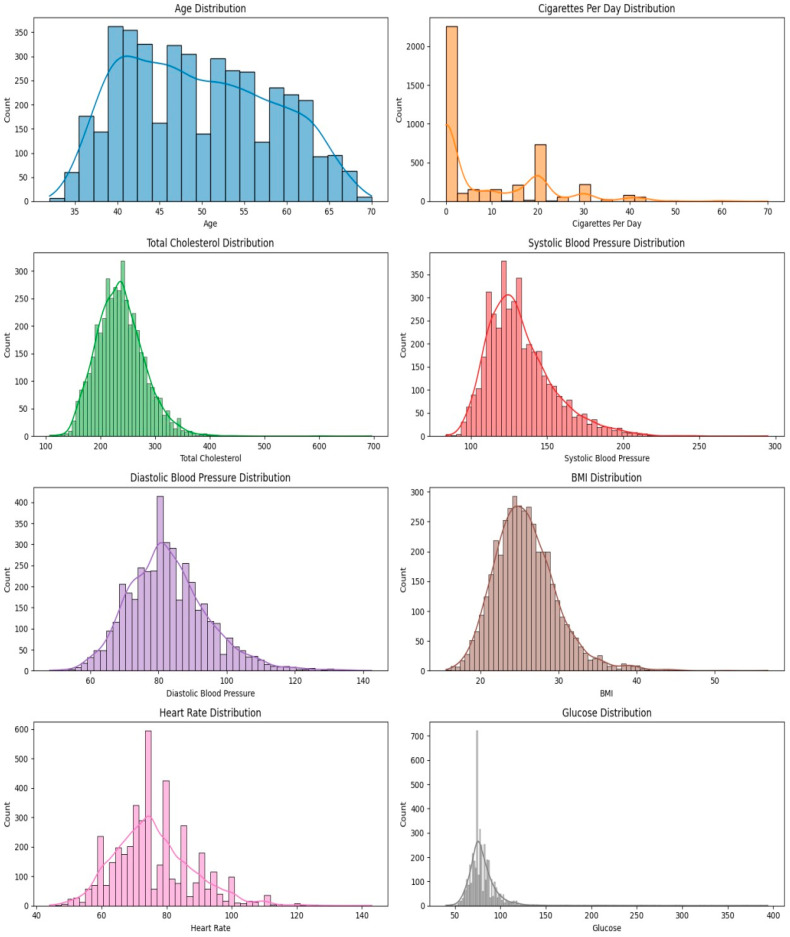
Distribution of key cardiovascular risk factors in the study population, including age, cigarettes per day, total cholesterol, systolic and diastolic blood pressure, body mass index (BMI), heart rate, and glucose for the Framingham dataset.

After identifying the Framingham dataset and the Cleveland subset of the UCI Heart Disease dataset as suitable public datasets for predicting coronary heart disease (CHD), we implemented a structured leakage-controlled machine learning pipeline consisting of data cleaning, stratified train-test splitting, training-data-only class balancing and feature scaling, model training, hyperparameter tuning, evaluation, and SHAP-based interpretability. Before model training, we performed data cleaning to ensure model input quality. In the Framingham dataset, several rows contained missing values across clinical features such as cholesterol or glucose levels. Rather than impute these values, which may introduce additional assumptions or clinical bias, we performed complete-case analysis by removing rows with missing data. Duplicate records were also checked and removed to prevent overrepresentation of any single patient. After complete-case filtering and duplicate removal, the Framingham dataset contained 3658 records with 15 clinical features and a binary target label, TenYearCHD, indicating whether the patient developed CHD within a 10-year period (Figure 1).

For the Cleveland subset of the UCI Heart Disease dataset, entries marked as “?” were treated as missing values, and incomplete records were removed. The disease outcome variable was binarized such that values greater than 0 were coded as heart disease-positive, and 0 as heart disease-negative. After cleaning, the Cleveland subset contained 297 records with 13 predictive features. We acknowledge that a complete-case analysis may reduce representativeness and introduce selection bias compared with imputation-based strategies. Therefore, this preprocessing choice is treated as a limitation of the current study rather than an optimized missing-data solution.

### 2.2. Class Balancing and Feature Scaling

**Figure 2:**
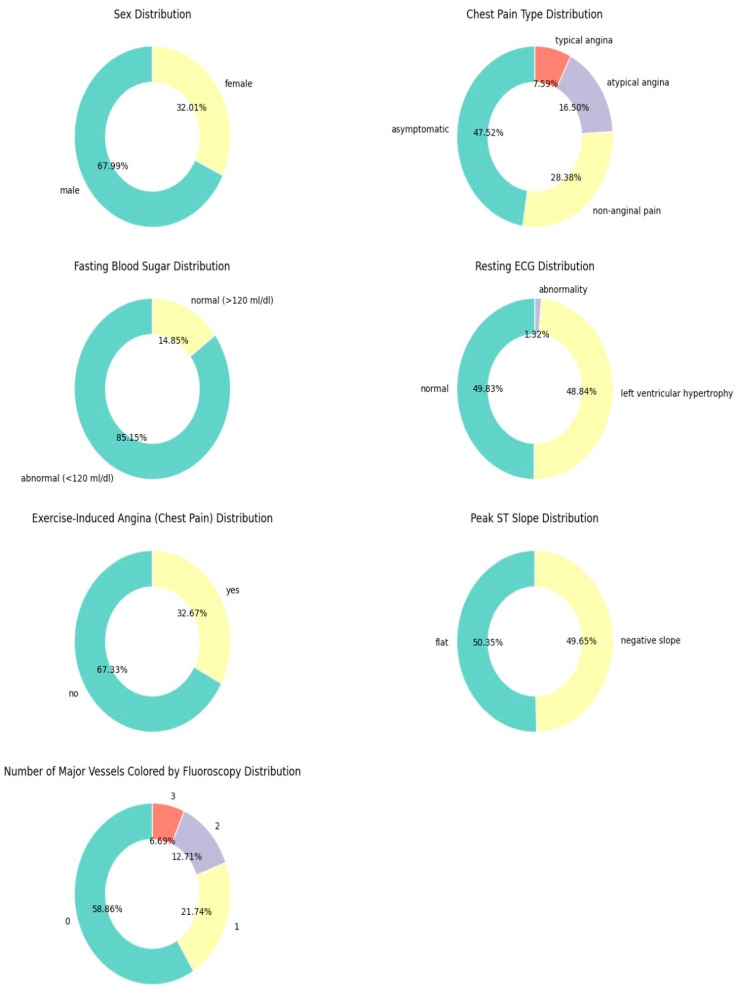
Distribution of categorical cardiovascular risk factors in the Cleveland subset of the UCI Heart Disease dataset (*n* = 303), including sex, chest pain type, fasting blood sugar, resting ECG findings, exercise-induced angina, peak ST slope, and the number of major vessels visualized by fluoroscopy.

Both datasets were imbalanced, with fewer positive heart disease cases than negative cases. This issue was particularly relevant for the Cleveland subset of the UCI Heart Disease dataset, which originally contained 303 records and was therefore more sensitive to sampling variability and class imbalance (Figure 2). To prevent data leakage, all preprocessing steps that could learn from the data distribution were performed only after the train-test split and only using the training data. Each dataset was split into stratified training and held-out test sets using an 80/20 split. For the Framingham dataset, the final complete-case analytic sample contained 3658 records, resulting in 2926 training samples and 732 held-out test samples. The positive-class rate was 15.2% in both the training and test sets. For the Cleveland subset of the UCI Heart Disease dataset, the final analytic sample contained 297 records after removal of incomplete entries, resulting in 237 training samples and 60 held-out test samples. The positive-class rate was 46.0% in the training set and 46.7% in the test set.

To address class imbalance, we used SMOTE (Synthetic Minority Over-sampling Technique) to generate synthetic minority-class samples, followed by random undersampling of the majority class to obtain an approximate 1:1 class ratio. Given a minority-class sample $x_{i}$ and one of its nearest minority neighbors $x_{zi}$, a synthetic sample $x_{new}$ is generated as

$$x_{new}=x_{i}+\lambda (x_{zi}-x_{i}),\lambda ∼U(0,1)$$

After resampling, features were standardized using z-score normalization:

$$z_{j}=\frac{x_{j}-\mu_{j}}{\sigma_{j}}$$

where ${\mu_{j}\;and\;\sigma }_{j}$ denote the mean and standard deviation of feature *j*, respectively. This standardization was especially important for algorithms sensitive to feature magnitude, such as K-nearest neighbors and neural networks.

### 2.3. Model Training and Evaluation

Five classifiers were evaluated: logistic regression, random forest, K-nearest neighbors, XGBoost, and a feedforward neural network implemented in PyTorch (2.12.0). Hyperparameter tuning for the classical machine learning models was performed using GridSearchCV with 5-fold cross-validation on the training data only. The classical models were trained and tuned using leakage-controlled imbalanced-learn pipelines, while the neural network was trained separately using PyTorch after training-set-only scaling and resampling. Final model evaluation was performed once on the untouched held-out test set.

Model performance was assessed using accuracy, precision, recall, F1-score, MCC, area under the receiver operating characteristic curve (AUROC), and Brier score. The Brier score was included as a probabilistic calibration metric, with lower values indicating better agreement between predicted probabilities and observed outcomes.

Best hyperparameter settings were: Framingham—logistic regression (C = 0.01, L2 penalty, liblinear solver), random forest (Gini criterion, maximum depth 8, square root feature sampling, 75 trees), K-nearest neighbors (Manhattan distance, 15 neighbors, uniform weighting), and XGBoost (column subsample 0.5, learning rate 0.01, maximum depth 3, minimum child weight 1, 50 estimators, subsample 0.5); Cleveland—logistic regression (C = 0.001, L2 penalty, liblinear solver), random forest (entropy criterion, maximum depth 12, square root feature sampling, 200 trees), K-nearest neighbors (Manhattan distance, 15 neighbors, distance weighting), and XGBoost (column subsample 0.5, learning rate 0.1, maximum depth 3, minimum child weight 3, 50 estimators, subsample 0.8).

### 2.4. Model Interpretability with SHAP

To improve interpretability, we used SHAP (SHapley Additive exPlanations), a post hoc explanation framework grounded in cooperative game theory. In SHAP, each feature is treated as a “player” in a cooperative game, and the model prediction is treated as the “payout” to be distributed according to feature contribution. For a model with *M* features, the prediction for an instance *x* can be expressed as an additive explanation model:

$$f(x)=\phi_{0}+\sum_{i=1}^{M}\phi_{i}$$

where $\phi_{0}$ is the expected model output over the background dataset and $\phi_{i}$ is the SHAP value for feature *i*, representing that feature’s contribution to shifting the prediction away from the baseline. The SHAP value for feature *i* is computed as the average marginal contribution of that feature across all possible feature subsets:

$$\phi_{i}=\sum_{S\subseteq F\setminus \{i\}}\frac{|S|!(M-|S|-1)!}{M!}\left[f_{S\cup \{i\}}(x_{S\cup \{i\}})-f_{S}(x_{S})\right]$$

where *F* is the full set of features and *S* is any subset not containing feature *i*. This formulation ensures that explanations satisfy local accuracy, consistency, and missingness. In this study, SHAP was applied to the XGBoost models as a consistent explanatory framework across datasets, yielding both global interpretability through mean absolute SHAP values and local interpretability through per-instance feature attributions.

## 3. Results

Performance was evaluated using accuracy, precision, recall, F1-score, MCC, AUROC, and Brier score. After rerunning the experiments using a leakage-controlled pipeline, performance estimates on the Framingham dataset were lower than in the preliminary analysis, indicating that the prior results were likely optimistic. On the Framingham dataset, logistic regression achieved the highest AUROC (0.7234), recall (0.6847), and F1-score (0.3938). Random forest achieved the lowest Brier score (0.1750), suggesting comparatively better probabilistic calibration, while the feedforward neural network achieved the highest accuracy (0.7719). However, positive-class precision remained low across all Framingham models, ranging from 0.2448 to 0.2872, reflecting the difficulty of predicting 10-year CHD outcomes in an imbalanced cohort.

**Figure 3:**
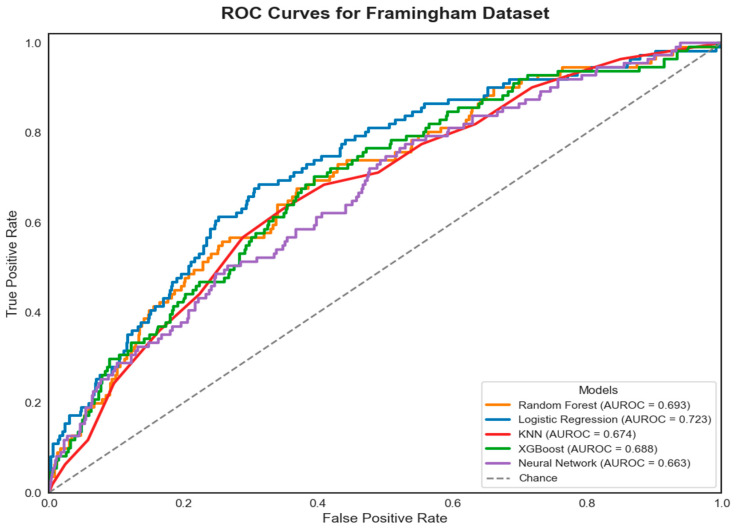
ROC curves comparing machine learning algorithms for predicting coronary artery disease using the Framingham dataset. AUROC values were 0.6929 for random forest, 0.7234 for logistic regression, 0.6741 for K-nearest neighbors, 0.6882 for XGBoost, and 0.6625 for the feedforward neural network.

**Figure 4:**
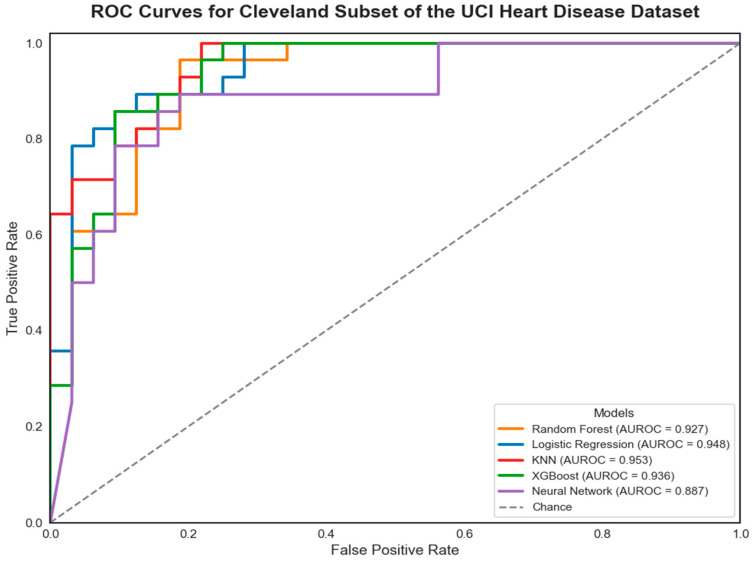
ROC curves comparing machine learning algorithms for predicting coronary artery disease using the Cleveland subset of the UCI Heart Disease dataset. AUROC values were 0.9275 for random forest, 0.9475 for Logistic Regression, 0.9531 for K-nearest neighbors, 0.9364 for XGBoost, and 0.8867 for the feedforward neural network.

On the Cleveland subset, logistic regression achieved the strongest threshold-based performance, with accuracy of 0.8667, precision of 0.8571, recall of 0.8571, F1-score of 0.8571, and MCC of 0.7321. K-nearest neighbors achieved the highest AUROC (0.9531) and the lowest Brier score (0.0942), indicating strong discrimination and favorable probabilistic calibration on the held-out test set. Random forest and XGBoost achieved similar accuracy values of 0.8333, while the feedforward neural network achieved competitive threshold-based performance but lower AUROC than the strongest classical models. Figure 3 and Figure 4 demonstrate the ROC curves for the two datasets.

## 4. Discussion

Reviewing the leakage-controlled analysis across the two datasets, our results suggest two main observational findings. First, model performance was strongly dataset-dependent, but the interpretation changed after correcting the preprocessing pipeline. In the Framingham cohort, performance was more modest than in the preliminary analysis, with logistic regression achieving the highest AUROC, random forest achieving the lowest Brier score, and the feedforward neural network achieving the highest accuracy. These findings suggest that prediction of 10-year CHD risk from the available Framingham variables is challenging under a strict held-out evaluation design, especially because the positive class is relatively rare (Table 2). In contrast, performance on the Cleveland subset of the UCI Heart Disease dataset remained stronger, with logistic regression achieving the best threshold-based metrics and K-nearest neighbors achieving the highest AUROC and lowest Brier score. However, these Cleveland results should be interpreted cautiously because the held-out test set contained only 60 patients (Table 3).

**Figure 5:**
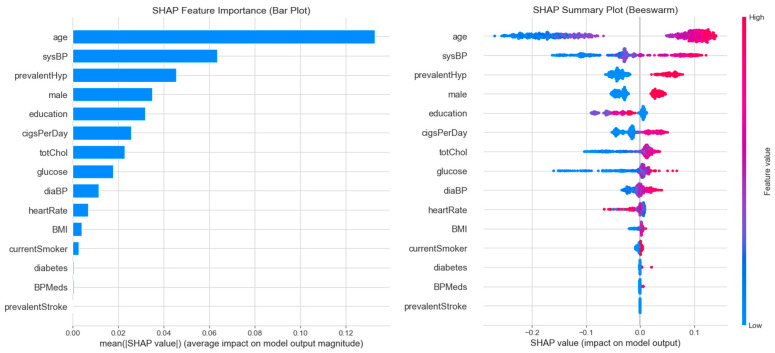
SHAP analysis using Framingham dataset of feature importance for the predictive model. (**Left**) Mean absolute SHAP values indicating the average impact of each feature on model output. (**Right**) SHAP summary plot showing the direction and magnitude of feature effects, with colors representing feature values from low (blue) to high (pink).

**Figure 6:**
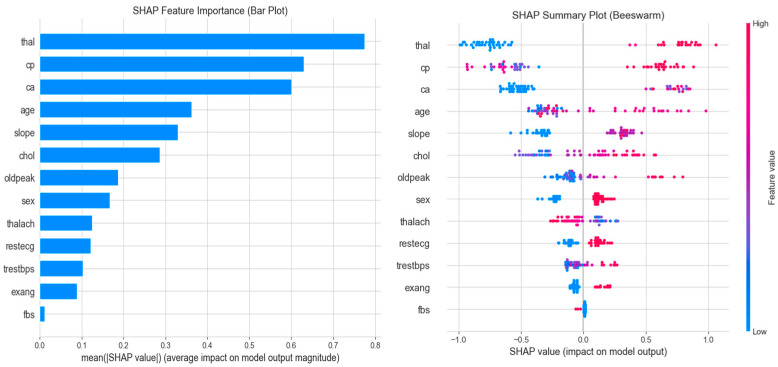
SHAP feature importance for the second predictive model using Cleveland subset of the UCI Heart Disease dataset. (**Left**) Mean absolute SHAP values indicating each feature’s average impact on model output. (**Right**) SHAP summary plot illustrates the direction and magnitude of feature contributions, with color gradients representing feature values from low (blue) to high (pink).

Second, SHAP enhanced explainability, bridging the gap between machine-learning predictions and clinical trust. Even when black box models were used, SHAP helped interpret how decisions were made and which features influenced them most. In the Framingham cohort, SHAP highlighted systolic blood pressure, smoking status, and hypertension as influential predictors, whereas in the Cleveland subset, number of major vessels, chest pain type, thallium stress test result (thal; normal, fixed defect, or reversible defect), and age were among the most influential features (Figure 5 and Figure 6). These identified predictors are broadly consistent with the current medical literature and best clinical screening practices [3,10,11]. Applying SHAP is therefore an important step in building clinical confidence in machine learning predictions and demonstrating that trained models prioritize features in ways that align with established cardiovascular knowledge.

As of 2025, machine learning models for cardiovascular disease prediction have become increasingly accurate, interpretable, and adaptable to clinical settings. Ensemble methods such as XGBoost, CatBoost, and LightGBM consistently achieve accuracies around 90 percent, with strong calibration and area-under-the-curve (AUC) values between 0.89 and 0.96, especially when combined with preprocessing techniques including feature scaling and SMOTE-based class balancing [8]. These models perform well with structured clinical data and effectively capture complex nonlinear relationships. Hybrid deep learning models, including convolutional neural networks combined with long short-term memory layers and attention-based architectures, can further improve performance. These models have demonstrated sensitivities and specificities above 97 percent and AUC values approaching 0.99 [12]. However, they require greater computational resources and are less transparent, limiting their clinical integration without additional interpretability. Transformer-based models such as TabPFN offer an efficient alternative, pretrained to generalize across tabular tasks and can deliver competitive performance on small- to medium-sized datasets with minimal tuning, making them practical for clinical research environments with limited computational resources [13]. Large language models such as AdaCVD extend predictive capability by incorporating structured variables and unstructured electronic health record data. AdaCVD has demonstrated strong generalizability across diverse patient populations and has outperformed traditional risk scores, especially in cohorts underrepresented in conventional datasets [14]. Privacy-preserving frameworks are also advancing, with federated learning approaches such as FedCVD++ enabling multi-institutional model training without requiring centralized data storage. This framework integrates logistic regression, support vector machines, neural networks, and tree-based models to achieve F1-scores comparable to or superior to centralized methods while addressing concerns related to data privacy and regulatory compliance [15].

The Framingham data were invaluable as a control cohort for comparing the efficacy of new medications, beta blockers, and ACE inhibitors [16]. One of the most valuable contributions was the demonstration that non-rheumatic atrial fibrillation was a strong risk factor for stroke and ischemic heart disease, leading to a flurry of controlled trials of newer classes of medications, including anticoagulants and antiarrhythmics, which are indispensable modern tools for managing heart disease [17]. Later cohorts recruited family members and descendants of the original participants, laying the groundwork for future identification of genetic risk factors [18].

Subsequent studies from the ongoing Framingham Heart Study identified additional cardiac risk factors, including increased left ventricular (LV) diameter, asymptomatic LV systolic dysfunction, diabetes, and hyperlipidemia, all of which remain highly relevant today [3]. Unfortunately, these features were not available in the open-source Framingham dataset, so our analysis focused on understanding the underlying methods utilized for complex medical datasets, learning to trace through historical documentation, and uncovering the innate difficulties entwined with medical machine learning.

A limitation of any healthcare project is the restricted availability of high-quality patient medical record data [19]. This project was only able to access the most well-known and well-studied cardiac datasets, which, unfortunately, limits our predictive scope to reproduce and validate prior feature correlation findings and test various machine learning models to try to optimize our model’s predictive ability. We were unable to find datasets that contained additional ancillary features collected from a patient’s medical record, which would have allowed us to perform feature extraction to identify any lesser-known CHD risk factors. Future studies should inspect comprehensive medical records to obtain the cleanest raw datasets and perform all preprocessing, standardization, and EDA from scratch. Having no control over the data collection, reporting, or documentation severely limits a researcher’s ability to draw novel conclusions from a historical retrospective dataset. Additionally, our research did not perform a target trial emulation, as current clinical trials are not focused on applications of machine learning [20]. While future utility exists in performing randomized clinical trials with machine learning, current ethical limitations have hindered progress. Several methodological limitations also remain. Model performance was evaluated using a single held-out test split rather than repeated cross-validation or bootstrap confidence intervals; therefore, the reported estimates may vary across different random splits, particularly for the smaller Cleveland subset. The Cleveland dataset contained only 60 held-out test samples after complete-case cleaning, limiting statistical stability and generalizability [21]. The Framingham analysis also relied on complete-case records, which may introduce selection bias relative to imputation-based strategies. Although the revised analysis addressed potential data leakage by performing train-test splitting before scaling, resampling, hyperparameter tuning, and final evaluation, and added Brier score assessment for probabilistic calibration, external validation on independent clinical cohorts remains necessary before any clinical deployment.

As machine learning research in the medical field advances, we are restricted to repeated analyses on the same handful of datasets, each with its own unique combination of inconsistencies, discrepancies, and duplications. Until open-source, high-quality medical datasets are made readily available outside the institutions that produce them, only researchers who align with the collecting institutions have unfettered access to identifiable patient information.

## 5. Conclusions

Importantly, the principal contribution of this work lies in demonstrating that model performance, probabilistic calibration, and interpretability are strongly dataset-dependent under a leakage-controlled evaluation framework. In the Framingham cohort, prediction of 10-year CHD risk remained challenging, with modest AUROC values and low positive-class precision across models. Logistic regression achieved the highest AUROC, random forest achieved the lowest Brier score, and the feedforward neural network achieved the highest accuracy. In contrast, the Cleveland subset showed stronger discrimination and threshold-based performance, with logistic regression achieving the best threshold-based metrics and K-nearest neighbors achieving the highest AUROC and lowest Brier score. However, these Cleveland results should be interpreted cautiously because of the small held-out test set. These findings reinforce the importance of cautious cross-dataset comparison, leakage-free preprocessing, Brier score-based calibration assessment, and external validation when developing machine-learning models for cardiovascular risk prediction.

Epidemiological cohort studies, like Framingham, contributed towards the shift in medical attitudes and perceptions of the time [22]. Rather than treating patients only after cardiovascular disease develops, the focus shifted toward preventing disease in high-risk populations and implementing early interventions to halt its progression. The quantification of various presentations and progressions of heart failure led to standardized assessments and diagnostic criteria, strengthening future data collection, analysis, and treatments.

We have concluded that the stark disparity between the wealth of medical data that exists in protected institutional systems, in comparison to de-identified limited data sets (LDS) available publicly, holds responsibility for limiting the pace of medical machine learning research at academic hospital institutions [23]. More effort should be placed on curating publicly available de-identified patient datasets, which could be split into healthy controls, grouped by disease, or have additional genomic information. Currently, this information is only accessible with institutional review board (IRB) approval, even from national consortium initiatives like the National Center for Biotechnology Information’s Database of Genotypes and Phenotypes (NCBI dbGaP). Reforming the space of medical data sharing to remain highly secure, yet able to disseminate bleeding-edge findings for open-source validation, could exponentially accelerate medical machine learning research, leading to improved health outcomes.

## Figures and Tables

**Table 1:** Structured comparison of prior cardiovascular machine learning studies and the present study.

Study	Dataset(s)	Models Evaluated	Interpretability	Calibration Metric	Reported Performance/Focus	Distinct Contribution
Yang and Guan, 2022 [7]	UCI Heart Disease	SMOTE-XGBoost	Limited/not central	Not emphasized	Feature optimization and SMOTE-XGBoost performance	Optimized a single model on UCI Heart Disease
Shah et al., 2025 [8]	Cardiovascular risk dataset	Hybrid ensemble ML	Explainable AI (XAI)	Discussed/reported	Strong ensemble-based cardiovascular risk prediction	Hybrid ensemble prediction with explainability
Yuda et al., 2025 [9]	Framingham Heart Study	ML models	Feature/risk-factor insights	Not central	Cardiovascular risk prediction and monitoring using Framingham data	Framingham-specific ML analysis
Bilal et al., 2025 [10]	Cardiovascular disease dataset	XAI-driven ML system	Explainable AI (XAI)	Not central	Precision forecasting using XAI	Explainable cardiovascular disease forecasting
Present study	Framingham + Cleveland subset of UCI Heart Disease	Logistic regression, random forest, KNN, XGBoost, feedforward neural network	SHAP applied consistently across both datasets	Brier score	Framingham: LR highest AUROC; RF lowest Brier score. Cleveland: LR best threshold metrics; KNN highest AUROC and lowest Brier score	Leakage-controlled cross-dataset comparison of discrimination, calibration, and SHAP-based interpretability

**Table 2:** Performance summary for Framingham dataset results.

Model	Accuracy	Precision	Recall	F1-Score	MCC	AUROC	Brier Score
Random Forest	0.7391	0.2872	0.4865	0.3612	0.2222	0.6929	0.1750
Logistic Regression	0.6803	0.2764	0.6847	0.3938	0.2698	0.7234	0.2138
K-Nearest Neighbors	0.6489	0.2448	0.6306	0.3526	0.2079	0.6741	0.2271
XGBoost	0.6899	0.2456	0.5045	0.3304	0.1762	0.6882	0.2255
Feedforward Neural Network	0.7719	0.2812	0.3243	0.3013	0.1664	0.6625	0.2048

**Table 3:** Performance summary for Cleveland subset of the UCI Heart Disease dataset results.

Model	Accuracy	Precision	Recall	F1-Score	MCC	AUROC	Brier Score
Random Forest	0.8333	0.8462	0.7857	0.8148	0.6652	0.9275	0.1122
Logistic Regression	0.8667	0.8571	0.8571	0.8571	0.7321	0.9475	0.2096
K-Nearest Neighbors	0.8333	0.8462	0.7857	0.8148	0.6652	0.9531	0.0942
XGBoost	0.8333	0.8750	0.7500	0.8077	0.6683	0.9364	0.1087
Feedforward Neural Network	0.8500	0.8276	0.8571	0.8421	0.6997	0.8867	0.1360

## Data Availability

The datasets analyzed in this study are publicly available. The Framingham Heart Study dataset and the Cleveland subset of the UCI Heart Disease dataset were obtained from publicly accessible sources cited in the manuscript. Processed data and code used for analysis are available from the corresponding author upon reasonable request.

## Supplementary Materials

The following supporting information can be downloaded at: https://www.mdpi.com/article/10.3390/epidemiologia7030075/s1, Table S1: Methodology for determining dataset evaluation Framingham Features; Table S2: Methodology for determining dataset evaluation UCI.

## Abbreviations

The following abbreviations are used in this manuscript:

AI	Artificial Intelligence
AUC	Area Under the Curve
CAD	Coronary Artery Disease
CatBoost	Categorical Boosting
CHD	Coronary Heart Disease
CHF	Congestive Heart Failure
CVD	Cardiovascular Disease
dbGaP	Database of Genotypes and Phenotypes
DL	Deep Learning
EDA	Exploratory Data Analysis
FFN	Feedforward Neural Network
F1-score	Harmonic Mean of Precision and Recall
GridSearchCV	Grid Search Cross-Validation
IHD	Ischemic Heart Disease
IRB	Institutional Review Board
KNN	K-Nearest Neighbors
LDS	Limited Data Sets
LightGBM	Light Gradient Boosting Machine
LV	Left Ventricle
ML	Machine Learning
MCC	Matthews correlation coefficient
ROC	Receiver Operating Characteristic
SHAP	SHapley Additive exPlanations
SMOTE	Synthetic Minority Oversampling Technique
SVM	Support Vector Machine
TabPFN	Tabular Prior-Data Fitted Network
TenYearCHD	10-Year Coronary Heart Disease Outcome
UCI	University of California, Irvine
XGBoost	Extreme Gradient Boosting
