# Supplementary material for: Machine Learning for Coronary Heart Disease Prediction: Comparative Analysis of Framingham and Cleveland Subset of the UCI Dataset with SHAP-Based Interpretability
Source: Epidemiologia (Basel). 2026 Jun 1;7(3):75. doi: 10.3390/epidemiologia7030075 (PMC13298444; doi:10.3390/epidemiologia7030075)
Supplement: Supplementary file 1 [file epidemiologia-07-00075-s001.zip › epidemiologia-4192953-supplementary.pdf]

Supplemental Files Index

S1: Methodology for determining dataset evaluation Framingham Features.

S2: Methodology for determining dataset evaluation UCI Data.

Exploratory Data Analysis

Known CAD Indicators (Advocate Healthcare, 2025):

Numerous risk factors for Coronary Artery Disease (CAD) have been identified, and advances in medical imaging have improved disease diagnosis and monitoring. For our predictive model, we focused on three categories of patient data: (a) biomarkers/labs, (b) clinical procedure reports, and (c) prior diagnoses. Standard labs include HDL, LDL, apoA-I, and HbA1c, while Troponins and D-dimer—although not routinely obtained—are strong indicators of cardiac injury when available. Clinical procedures such as ECG, echocardiography, catheterization, and CT/MRI imaging provide valuable insight, but only numeric Stress Test data (e.g., maximum heart rate achieved) were incorporated due to the complexity of image labeling. Prior history of myocardial infarction, ischemia, aneurysm, or arrhythmias also serves as a strong predictive feature.

For model development, we evaluated three primary datasets: (A) CDC BRFSS, (B) UCI Heart Disease, and (C) Framingham Heart Study. BRFSS provides extensive self-reported survey data (>400,000 adults annually), but its size and subjectivity made it unsuitable for our computational capacity. The UCI dataset (1,190 patients) is widely cited but plagued by duplicates, missing features, and poorly documented preprocessing, leaving the 303-patient Cleveland subset as the only reliable source. The Framingham Heart Study—a foundational, longitudinal cohort beginning in 1948—identified hypertension, rheumatic heart disease, and prior CHD as major predictors of congestive heart failure, and introduced the now-standard Framingham Risk Score.

After dataset evaluation, we selected the Cleveland and Framingham datasets for training and testing. Five machine learning models were developed: Logistic Regression, Random Forest, K-Nearest Neighbors (KNN), XGBoost, and a Feedforward Neural Network (FFN) to predict CAD risk and progression.

Framingham Data Dictionary

Table S1. Framingham Features (15 + 1 Target).

	Feature Name (label)	DescriptiveName	Description	Raw Coding	Definitions
1	**sex**	Sex	sex of the patient	[1: Male, 0: Female]	
2	**age**	Age	age of the patient	[years]	
3	**education**	Education	Educational level of patient	[0-4]	Not originally collected, no metadata mapping labels.
4	**currentSmoker**	Current Smoker		0: no smoker; 1: yes smoker	
5	**cigsPerDay**	Cigarettes per Day	Number of Cigarettes Smoked per Day		
6	**BPMeds**	Blood Pressure Medication	If patient is taking any hypertensive medications	0: no; 1: yes	
7	**prevalentStroke**	Stroke	Any history of Stroke (recorded or diagnosed)		

8	**prevalentHyp**	Hypertension (high blood pressure)	Diagnosed by<br>1.) abnormal BP on exam;<br>2.) Taking anti-hypertensive medications		
9	**diabetes**	Diabetes mellitus	Diagnosed by<br>1.) blood glucose > 150 mg/100mL;<br>2.) receiving treatment for Diabetes;<br>3.) Record of diagnosis	0: no;<br>1: yes	
10	**totChol**	Cholesterol	serum cholesterol	[mm/dl]	(millimeters per deciliter)
11	**sysBP**	Systolic BP	Force from heart squeezing		BP Numerator
12	**diaBP**	Diastolic BP	Force from heart at rest (to prevent blood backflow)		BP Denominator
13	**BMI**	Body Mass Index	Calculated BMI	Float	
14	**heartRate**	Heart Rate	heart rate per minute, recorded by ECG		
15	**glucose**	Blood Glucose levels	Fasting blood glucose	[mg/100 mL]	

16	**TenYearCHD**	Presence of Coronary Heart Disease	Target Outcome	0: no CHD; 1: yes CHD	Qualifying Events: myocardial infarction, coronary insufficiency, angina pectoris, sudden death from CHD, non- sudden death from CHD

Cleveland Data Dictionary

Table S2. *UCI Features (13 + 1 Target).*

	Feature Name (label)	Descriptive Name	Description	Raw Coding	Definitions
1	**age**	Age	age of the patient	[years]	
2	**sex**	Sex	sex of the patient	[1: Male, 0: Female]	
3	**cp**	ChestPainType	chest pain type	[0: Typical Angina;<br>1: Atypical Angina;<br>2: Non-Anginal Pain;<br>3: Asymptomatic]	

4	**trestbps**	RestingBP	resting blood pressure (on admission to hospital)	[mm Hg]	(millimeters of mercury)
5	**chol**	Cholesterol	serum cholesterol	[mm/dl]	(millimeters per deciliter)
6	**fbs**	Fasting Blood Sugar	fasting blood sugar	[1: if FastingBS > 120 mg/dl, 0: otherwise]	
7	**restecg**	RestingECG	resting electrocardiogram results	[0: Normal;<br>1: having ST-T wave abnormality (T wave inversions and/or ST elevation or depression of > 0.05 mV);<br>2: showing probable or definite left ventricular hypertrophy by Estes' criteria]	The Romhilt-Estes (RE) score assigns points for the presence of certain ECG findings, and a score of 4 is considered probable LVH, while a score of 5 or greater indicates definite LVH. Left ventricular hypertrophy (LVH) means the muscle of the heart's main pump (left ventricle)

					has become thick and enlarged.
8	**thalach**	MaxHR	maximum heart rate achieved during Stress Test	[Numeric value between 60 and 202]	
9	**exang**	ExerciseAngina	exercise-induced chest pain	[1: Yes, 0: No]	
10	**oldpeak**	Oldpeak	oldpeak = ST depression induced by exercise relative to rest	[Numeric value measured in depression]	ST depression induced by exercise relative to rest

11	**slope**	ST_Slope	the slope of the peak exercise ST segment	[Up: upsloping; Flat: flat;<br>Down: downsloping]	
12	**ca**	NumVessels	Number of major vessels colored by Fluoroscopy	[0-3]	Use continuous X-rays and contrast dyes to visualize how blood flows (or does not flow) through vessels
13	**thal**	Congenital	Normal or abnormal heart	3 = normal; 5 = fixed defect; 7 = reversible defect	
14	**num**	HeartDisease	Target outcome	[1: heart disease, 0: Normal]	Value 0: < 50% diameter narrowing;<br>Value 1: > 50% diameter narrowing (stenosis)

After analyzing both the Framingham and Cleveland datasets, and grouping them by the target outcome of CHD, we can see that both show similar skewed distribution patterns towards healthy patients and bias towards male patients, although the Cleveland dataset contains far more positive cases (Figures 3 and 6). For overlapping features between both datasets (Sex, Age, Smoking, etc), we can see similar distribution patterns and features captures, suggesting the results of one model may be generalizable to the other.
